# Effect of Intravitreal Conbercept Injection on Complications of Pars Plana Vitrectomy in Patients with Proliferative Diabetic Retinopathy

**DOI:** 10.3390/jpm13040572

**Published:** 2023-03-23

**Authors:** Yuzhi Ding, Na Su, Jie Luan, Yan Ni, Zilin Sun

**Affiliations:** 1 Department of Ophthalmology, Zhongda Hospital Southeast University, Nanjing 210009, China; 2 Department of Ophthalmology, The First Affiliated Hospital of Nanjing Medical University, Nanjing 210029, China; 3 Department of Endocrinology, Zhongda Hospital, Institute of Diabetes, School of Medicine, Southeast University, Nanjing 210009, China

**Keywords:** proliferative diabetic retinopathy, anti-VEGF drugs, pars plana vitrectomy, VEGF-A, vitreous hemorrhage

## Abstract

Background: The effect of intravitreal conbercept (IVC) before pars plana vitrectomy (PPV) on surgical complications in patients with proliferative diabetic retinopathy (PDR) was observed. Methods: A total of 152 patients with PDR operated on in Jiangsu Provincial People’s Hospital from November 2019 to November 2020 were divided into two groups: 124 patients in the preoperative intravitreal conbercept injection + PPV group (IVC group) and 28 patients in the PPV only group (No-IVC group). Vitreous samples were collected in all eyes of patients who underwent vitrectomy, and the content of VEGF-A was measured by Luminex. The effect of conbercept on intraoperative and postoperative complications of PDR was assessed. Results: The content of VEGF in the vitreous of the IVC group was significantly lower than that in the No-IVC group (64.50 ± 58.40 pg/mL vs. 805.17 ± 417.60 pg/mL, *p* < 0.001). During postoperative follow-up, early postoperative vitreous hemorrhage (VH) occurred in 13 of 142 eyes (9.15%). Compared with the No-IVC group, PDR patients with VH and fibrovascular membrane (FVM) or high complexity in the IVC group had lower intraoperative bleeding rates (*p* < 0.05). The early postoperative hemorrhage rate in the IVC group was lower than in the No-IVC group (6.03% vs. 23.08%, *p* < 0.05). The number of intraoperative electrocoagulation and iatrogenic retinal holes in the IVC group was significantly lower than in the No-IVC group (*p* < 0.05). There were no significant differences in intraocular hypertension and NVG numbers between the two groups. Visual acuity in both groups improved after PPV surgery, reaching the highest level in the 3rd month after the operation. Conclusions: IVC before PPV can reduce the level of VEGF-A in the vitreous body and reduce surgical complications.

## 1. Introduction

Diabetic retinopathy (DR), with a prevalence of 34.6%, is a common microvascular complication of diabetes. The late stage of DR is proliferative diabetic retinopathy (PDR), which accounts for 6.96% [1]. Studies have found that pathological neovascularization is related to the increase in VEGF level in the vitreous, which is the main pathological feature of PDR. High VEGF levels may be associated with retinopathy and promote intraoperative or postoperative complications [2,3,4]. The breakdown of the blood–retinal barrier and the lack of structural integrity leads to protein and fluid leakage and vitreous hemorrhage (VH), which promotes fiber proliferation and even leads to complications such as traction reticulum detachment (TRD), neovascular glaucoma (NVG) and persistent macular edema, leading to severe vision loss and even blindness [5]. Pars plana vitrectomy (PPV) should be considered when VH is not absorbed or fibrovascular membrane (FVM) traction causes retinal holes and TRD. There are two types of PDR patients proposed for PPV surgery: VH and VH + FVM. The operation of PDR with FVM is relatively complicated. Bleeding often occurs during the removal of FVM, which affects the surgical field, increases the operation difficulty, and finally leads to an increase in intraoperative and postoperative complications [6]. Some scholars have proposed that the preoperative injection of anti-VEGF drugs can significantly reduce the intravitreal VEGF level and inhibit neovascularization and vessel leakage, thereby decreasing the incidence rate of intraoperative bleeding, postoperative bleeding, macular edema, to effectively improve the prognosis of PDR surgery [7,8].

Early postoperative complications were defined as complications within 1 month, and late postoperative complications occurred 1 month later [9,10,11]. The main postoperative complications observed included high intraocular pressure, NVG, and postoperative rebleeding. However, whether anti-VEGF drugs can reduce early or late postoperative bleeding is still inconsistent [7,10,12]. Conbercept is a recombinant fusion protein that acts as a multitarget inhibitor of the VEGF family. It can effectively bind to VEGF-α, VEGF-β and placental growth factors. Animal studies and clinical trials have shown that conbercept can significantly inhibit angiogenesis, inflammation and oxidation [13,14]. In this study, intravitreal conbercept injection was performed 1 week before PPV. The relationship between anti-VEGF drug treatment and the incidence rate of PPV complications was observed to provide a reference for the prevention and treatment of hemorrhage after PPV.

## 2. Materials and Methods

### 2.1. General Information

A total of 152 patients with PDR admitted to Jiangsu Provincial People’s Hospital from November 2019 to November 2020 were selected, including two types: pure VH and VH + FVM. In addition, 17 patients with macular holes without other eye diseases were selected as the control group to compare the VEGF levels in the vitreous. Inclusion criteria: (1) age > 18 years old; (2) poor absorption of VH (>1 month) or FVM traction to form rhegmatogenous or TRD; (3) voluntarily participate in this study and sign the informed consent form. Exclusion criteria: (1) previous vitrectomy; (2) previous anti-VEGF therapy; (3) other eye diseases affecting the observation of indicators. The patients were divided into the IVC group (*n* = 124) and the No-IVC group (*n* = 28). There was no significant difference in average age, duration of diabetes, duration of PDR, BCVA, and other general data between the two groups (Table 1), which were comparable between the two groups (*p* > 0.05). All subjects gave informed consent for inclusion before they participated in the study. The study was conducted in accordance with the Declaration of Helsinki, and the protocol was approved by the Ethics Committee of Jiangsu Provincial People’s Hospital (2017-SR-283). CONCEPT of clinical trials has been registered at https://clinicaltrials.gov/ (ID NCT03506750, accessed on 24 April 2018). The patients included in this study were randomly assigned according to the CONCEPT clinical trials.

### 2.2. Treatment

One week before PPV, patients in the IVC group were treated with intravitreal injections of conbercept (10 mg/mL, 0.05 mL, Chengdu Kanghong Pharmaceutical Group Co., Ltd., Chengdu, China), and others in the No-IVC group did not receive any treatment. Both groups underwent minimally invasive 23 G vitrectomy: after routine disinfection and towel, 4 mL Ropivacaine Hydrochloride Injection was used for retrobulbar anesthesia. At the corneoscleral margin of 3.5 mm, 15° to 30° parallel to the scleral margin, the Entry System was performed in the superior temporal, inferior temporal, and superior nasal directions, and the 6 mm cannula was retained. The perfusion tube was inserted into the cannula and ensured the tube head was in the vitreous. The perfusion was temporarily closed until the vitreous fluid was collected. A Vitreous Cutter, Canaloplasty microcatheter and other intraocular instruments were used to perform vitrectomy through an indwelling cannula above the nose. PPV was carried out along with VH removal, proliferative membrane stripping, intraocular electrocoagulation, retinal photocoagulation, and silicone oil or air filling according to the disease condition. At the end of the operation, the cannula and the perfusion tube were pulled out, the incision was gently pushed in the reverse puncture direction with a wet cotton swab, and the sclera incision was slightly compressed and closed. At the end of the surgery, Tobramycin and Dexamethasone were applied to the conjunctival sac of the operated eye. Postoperatively, Prednisolone Acetate Ophthalmic Suspension and Ofloxacin Eye drops were used for 1 month. The No-IVC and control groups were treated with PPV alone, and cataract phacoemulsification combined with intraocular lens implantation should have been performed at the same time if the cataract affected the intraocular operation.

### 2.3. Observational Outcomes

The number of patients, average age, duration of DR, duration of PDR, BCVA, and the content of VEGF in vitreous were recorded before the operation. Baseline fundus photography and OCTA images of the two groups were collected, as well as OCTA images of the IVC group after anti-VEGF treatment, while OCTA images of the No-IVC group were taken at the same time for comparison. During the operation, the bleeding, the electrocoagulation times, the number of iatrogenic holes, and the operation time were recorded. Early postoperative complications were defined as complications within 1 month, and late postoperative complications occurred 1 month later [9,10,11]. Visual acuity, intraocular pressure (IOP), and rebleeding were observed 1 week, 1 month, and 3 months after surgery. The incidence of NVG was followed up two years after surgery. In this study, intraoperative complication assessment was performed by an associate chief physician who was familiar with the study design and acted as an intraoperative assistant. The record of surgical complications was completed by a resident physician. BCVA was measured by the same experienced optometrist using the Snellen chart. For the convenience of statistics, the results were converted into Log MAR visual acuity for analysis.

There are two ways to determine intraoperative bleeding:

(1) The intraoperative hemorrhage score (HS) was recorded: 0: no bleeding; 1: mild bleeding, which can be stopped spontaneously or by increasing perfusion pressure; 2: moderate bleeding, requiring electrocoagulation; 3: a large amount of bleeding, covering more than half of the area of the posterior pole.

(2) Score based on complexity score (CS): According to the Castellarin A [11] rule, PDR is divided into two groups: SC score < 3 and SC score ≥ 3. ① The number of quadrants of fibrovascular proliferation (FVP) (CS in each quadrant was marked as 1 point, a total of 4 quadrants); ② the position of FVP: before the equator (0 points), after the equator (0 points), and before and after the equator (1 point); ③ TRD (1 point); ④ traction rhegmatogenous retinal detachment (2 points); ⑤ posterior vitreous detachment (No, 1 point).

### 2.4. Vitreous Fluid Collection and Determination of VEGF Content

Before vitrectomy, without turning on the perfusion switch, a 2 mL sterile air syringe was used to drain the remaining test fluid in the vitrectomy tube. About 0.5 mL of vitreous was cut and aspirated without perfusion. A centrifuge at 1000 rpm was used to remove cells and blood, and the supernatant was stored in sterile tubes and immediately stored at −80 °C for measurement within 6 months after collection. Our previous study suggested that two diseases, idiopathic macular epiretinal membrane and macular hole (MH), could be combined as a control group for PDR [15]. The human VEGF-A content was determined using a liquid chip commercial kit (Cytometric Bead Array Flex Kit; Becton, Dickinson and Company, Franklin Lakes, NJ, USA). Two-color flow cytometry analysis was performed using flow cytometry (FACSCalibur; Becton, Dickinson and Company, Frankelin Lakes, NJ, USA). For VEGF-A, the lowest detectable concentration for this assay was 4.5 pg/mL. Any concentration below this level was recorded as the lowest detectable concentration for statistical analysis.

### 2.5. Statistical Methods

SPSS 19.0 software was used to analyze the data, and the measurement data were expressed as mean ± standard deviation ($\bar{x}$ ± S). The *t* test was used for comparison between groups, and the paired *t* test was used before and after treatment in each group. Count data were expressed as frequency, and the chi-square test (χ^2^) or exact *t* test was used to compare the rates. *p* < 0.05 was considered statistically significant.

## 3. Results

### 3.1. Comparison of Baseline Data between the Two Groups

The baseline characteristics of the two groups are summarized in Table 1. There was no significant difference in age, duration of disease, and visual acuity between the two groups (*p* > 0.05), which were comparable. The duration of diabetes in each group was about 10 years or more, and PDR duration, a common microvascular complication of diabetes, was about 3 months.

### 3.2. VEGF-A Levels

**Figure 1:**
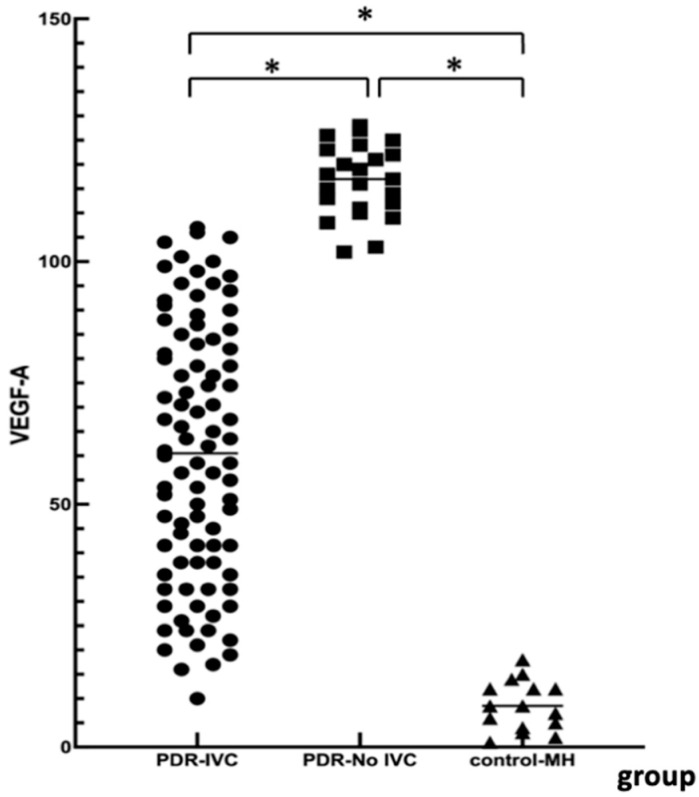
VEGF-A level in vitreous cavity between different groups. PDR: proliferative diabetic retinopathy; PDR-IVC: PDR patients with intravitreal injection of conbercept + pars plana vitrectomy; PDR-No-IVC: PDR patients with pars plana vitrectomy only; Control-MH: control group was patients with macular holes. * *p* value < 0.05.

The VEGF level in the IVC group was significantly lower than that in the No-IVC group (*p* < 0.001) and higher than that in the control group (Table 2, *p* < 0.001). The intravitreal injection of 0.05 mL of conbercept reduced vitreous VEGF levels in PDR patients, but compared with the control group, the level remained higher. This is also PDR patients get anti-VEGF drugs more than once. The VEGF-A concentration in the vitreous cavity between different groups is shown in Figure 1.

### 3.3. Fundus Images (Figure 2)

**Figure 2:**
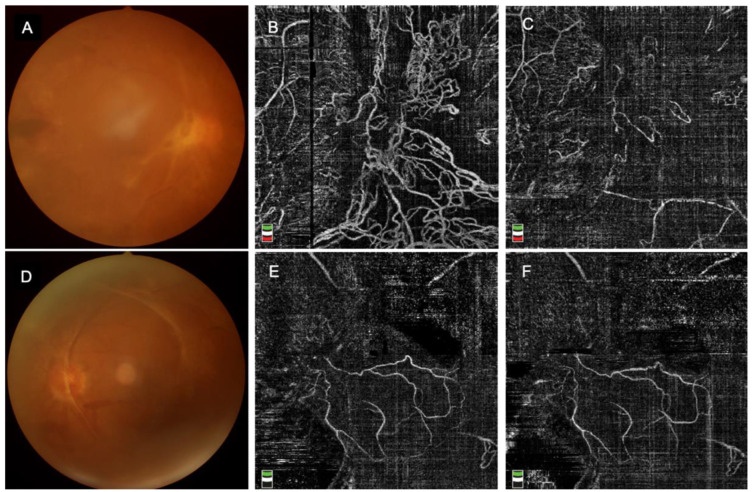
Images of the representative cases with and without IVC, (**A**). Fundus photograph before IVC, (**B**). OCTA image before IVC, (**C**). OCTA image after IVC, (**D**). Fundus photograph in the No-IVC group, (**E**,**F**). In the No-IVC group, OCTA images scanned at the same time as patients in the IVC group.

Before PPV surgery, fundus images were taken for each participant to present the fundus condition. As shown in Figure 2, after intravitreal conbercept injection, the choroidal neovascularization(CNV) decreased significantly in the IVC group, while in the No-IVC group, it had no significant change. Anti-VEGF treatment immediately reduces CNV and thus inhibits proliferation.

### 3.4. Surgery-Related Bleeding

According to the intraoperative hemorrhage score (HS) recorded during surgery (Table 3), all PDR patients were consistent at the level of mild to moderate bleeding. In the IVC group, 26 of 124 PDR patients with VH experienced no bleeding or mild bleeding, as with the No-IVC group. For patients with VH and FVM, the HS in the IVC group was significantly lower than that in the No-IVC group (*p* < 0.05); that is, the intravitreal injection of conbercept reduced surgery-related bleeding in PDR patients with VH and FVM.

According to the intraoperative bleeding score based on complexity score (SC) in Table 4, in the SC < 3 subgroup, the surgery hemorrhage rate had no statistical difference between the IVC group and the No-IVC group. However, in the SC ≥ 3 subgroup, compared with that in the No-IVC group, the surgery hemorrhage rate in the IVC group significantly reduced. This showed that, especially in complex PDR, patients benefit from intravitreal conbercept treatment.

As shown in Table 5, the postoperative rebleeding rate at 1 month in the IVC group (6.03%) was significantly lower than that in the No-IVC group (23.08%, *p* = 0.012), while there was no significant difference between the two groups in the late postoperative period (3 months, *p* = 0.361).

### 3.5. Intraocular Pressure and NVG

Stable intraoperative and postoperative intraocular pressure (IOP) is important for patients undergoing PPV. NVG occurred in patients with ocular hypertension. In the IVC group, 70 cases were filled with silicone oil and 54 cases with gas (air or C3F8) during vitrectomy. In the No-IVC group, 16 cases were filled with silicone oil and 12 with air. For early and late high IOP rates, it was kept at a low level in both groups, and there was no significance between the two groups (Table 6, *p* > 0.05). During the postoperative 2-year follow-up, the number of patients with NVG was 13 in the IVC group and 2 in the No-IVC group, with no significant difference between the groups (Table 7, *p* > 0.05).

### 3.6. Other Secondary Outcomes (Table 8)

The operation time was similar between the two groups (IVC group vs. No-IVC group, 37.86 ± 30.22 min vs. 35.21 ± 23.97 min, *p* > 0.05). The amount of electrocoagulation in the IVC group was less than that in the No-IVC group (1.12 ± 1.40 vs. 1.78 ± 1.72, *p* < 0.05), especially in VH + FVM (1.23 ± 1.49 vs. 2.53 ± 1.60, *p* < 0.01). The number of iatrogenic holes in the IVC group was less than that in the No-IVC group (0.35 ± 2.33 vs. 0.48 ± 2.24, *p* < 0.05), especially in VH + FVM (0.45 ± 1.53 vs. 0.89 ± 1.60, *p* = 0.01). During the follow-up of PDR patients, visual acuity improved in all groups except the No-IVC group at 1 month, and there was no significant difference between the groups (*p* > 0.05).

**Table 8:** BCVA changes between two groups.

Group	Preoperative		1 Week		1 Month		3 Month	
	*n*	BCVA	*n*	BCVA	*n*	BCVA	*n*	BCVA
IVC	124	1.67 ± 0.97	117	1.08 ± 0.97	116	0.77 ± 0.78	109	0.60 ± 0.77
No-IVC	28	1.65 ± 0.84	25	1.20 ± 0.83	26	1.95 ± 3.47	25	0.64 ± 0.81
*p* value	—	>0.05	—	0.73	—	0.29	—	0.901

IVC: Intravitreal conbercept injection + pars plana vitrectomy; No-IVC: pars plana vitrectomy only; BCVA: best-corrected visual acuity.

## 4. Discussion

The changes in the retinal microenvironment, such as chronic high glucose toxicity and hypoxia in PDR, lead to the damage of retinal vascular endothelial cells; stimulate the synthesis and secretion of VEGF in retinal pericytes, endothelial cells, retinal pigment epithelium, and other cells; increase vascular permeability; and induce pathological angiogenesis. Pathological neovascularization has brittle vessel walls and can easily cause VH. The damage of the blood–retinal barrier and the lack of structural integrity of neovascularization can cause protein and fluid leakage and hemorrhage, promote fiber proliferation and even cause TRD, NVG and persistent macular edema, which are the main causes of blindness in diabetes [5,16]. At present, preoperative adjuvant anti-VEGF therapy can reduce intraoperative bleeding, intraoperative electrocoagulation times and the probability of iatrogenic holes, shorten the operation time, reduce the risk of early postoperative bleeding, and improve the visual prognosis of PDR. Still, it may only be effective for complex PDR, and the benefit of patients with simple VH is limited [17]. Evidence from this study suggests that the use of anti-VEGF before PPV can significantly reduce the probability of intraoperative and postoperative bleeding in complex PDR patients with FVM, and this phenomenon can be explained by the level of VEGF-A in the vitreous.

Conbercept is a recombinant fusion protein that can reduce the levels of VEGF-A in the eye, induce the apoptosis of vascular endothelial cells and the occlusion of neovascularization, reduce fundus hemorrhage, maintain the stability of the blood–retinal barrier, and prevent the occurrence of postoperative complications such as VH [18,19,20]. This study is consistent with the results reported by Gao S [21], Su L [22], and Bing Li [23]. IVC before PPV is safe and effective, the adhesion between FVM and retina is reduced, and intraoperative bleeding is decreased. According to the comparison of “complexity score” [11], our study revealed that intravitreal conbercept injection in complicated PDR surgery always leads to a lower bleeding rate. This is different from the conclusion of Gao S [21] that there is no difference between the intraoperative bleeding rate and the complexity of PDR. It is possible that PDR with high complexity has a multi-quadrant proliferative membrane. After IVC treatment, the FVM is constricted to reduce bleeding. However, the inconsistent conclusions in different studies may be related to the baseline data of patients and the severity of the disease. Postoperative VH is the most common complication after PPV in PDR patients, with an incidence ranging from 6% to 75% [24]. Our results showed a significantly lower rate of VH at 1 month after surgery in the IVC group, consistent with the finding that VEGF levels were lower at 1 month [25]. Gao S’s study [21] pointed out that the bleeding rate had no significant difference both in the early and late postoperative periods when PDR patients recieved an intravitreal injection of conbercept before, during, or after surgery. However, in the present study, the bleeding rate in the IVC group was lower than that in the No-IVC group. This may have been due to the influence of anti-VEGF drugs, but we cannot rule out the possibility that the inconsistent results were caused by different experimental designs, operators, and baseline data. More comprehensive clinical trials are needed to verify the results in the future. During surgery, the number of electrocoagulation and iatrogenic holes in the IVC group were significantly lower, especially in VH + FVM patients, and there was also no significant benefit for PDR patients with simple VH.

Elevated VEGF-A levels are associated with retinal neovascularization and disease complexity. We measured VEGF levels in the PDR and the control groups. There were significant differences among the three groups. VEGF-A was significantly higher in the PDR group than in the simple MH group. VEGF-A levels in the No-IVC group were significantly higher than in the IVC group. This further demonstrated the necessity of preoperative anti-VEGF treatment in PDR patients with VH + FVM. However, in PDR patients with simple VH, anti-VEGF treatment may have limited benefits. During postoperative follow-up, the visual acuity in the PDR groups improved, and BCVA at 3 months was consistent with the results of Ren [18]. In the No-IVC group, the worst visual acuity was consistent with a high rebleeding rate 1 month after surgery. One patient in the IVC group had rebleeding and improved visual acuity from 0.3 to 0.6 after a second surgery. In the No-IVC group, three cases had rebleeding, and one had improved visual acuity from counting fingers to 0.4 after the second surgery. The visual acuity of two patients did not improve after the second operation, and the visual acuity remained in hand motion. There were no cases of blindness after multiple surgeries. Thus, conbercept effectively inhibited neovascularization and promoted complete FVM stripping in the IVC group, therefore preventing early postoperative VH.

It is known that intraoperative neovascularization or FVM rupture can cause bleeding, but the source of postoperative VH is still difficult to determine. Some scholars have pointed out that the early postoperative VH is related to several possible factors, such as FVM removal, the reactivation of residual FVM in the retina, the bleeding of the damaged retinal tissue, the inflammation caused by the surgical trauma, and the increase in VEGF concentration [26]. Late VH is considered to be related to recurrent neovascularization caused by a combination of factors such as blood glucose control, disease development, the retention time of anti-VEGF drugs, and the adequacy of PRP [27]. Therefore, scholars have also put forward unique opinions on how to effectively prevent postoperative hemorrhage: high intraocular VEGF levels in PDR patients during initial PPV have been identified as an important risk factor for early postoperative hemorrhage [25]. In addition, to minimize surgical trauma, operators usually remove the residual FVM as much as possible during PPV surgery, and VEGF concentration measurement in the vitreous before PPV can be used as a biomarker [28]. According to Du L [29] and HU Z [30,31], IVC has a significant inhibitory effect on neovascularization 3–7 days before PPV. It can avoid the occurrence of TRD caused by excessive fibrosis and remove the FVM as much as possible during the operation to prevent postoperative VH or NVG.

This study still has the following limitations: The study was a post hoc study. The sample size was relatively small, the follow-up time was short, and the personnel in each follow-up were different. These defects may have led to bias or errors in the experimental results. Despite these limitations, the study supports the association of VEGF levels with surgical outcomes in PPV and the safety and efficacy of adjuvant conbercept in the treatment of PDR before PPV. Anti-VEGF therapy can decrease the level of VEGF in the vitreous of patients with complex PDR, safely and effectively reduce the intraoperative and early postoperative bleeding, reduce the occurrence of intraoperative electrocoagulation and iatrogenic hole, and improve the efficiency of surgery.

## Figures and Tables

**Table 1:** Comparison of preoperative baseline data ($\bar{x}$ ± s).

	Number (Female)	Age (y)	PDR Duration (m)	Duration of Diabetes (y)	BCVA (Log MAR)	Complexity Score > 3
IVC	124 (76)	50.62 ± 11.72	3.42 ± 4.54	9.84 ± 7.55	1.67 ± 0.97	42 (33.87%)
No-IVC	28 (16)	55.17 ± 9.52	3.36 ± 3.14	13.00 ± 10.09	1.65 ± 0.84	10 (35.71%)
*p*	>0.05	>0.05	>0.05	>0.05	>0.05	>0.05

PDR: proliferative diabetic retinopathy; BCVA: best-corrected visual acuity; Log MAR: logarithm of the minimum angle of resolution.

**Table 2:** VEGF-A level in vitreous between different groups ($\bar{x}$ ± s).

Group (*n*)	VEGF-A (pg/mL)	*p* Value
PDR-IVC (124)	64.50 ± 58.40	0.000 *
PDR-No-IVC (28)	805.17 ± 417.60	
Control-MH (17)	20.63 ± 12.12	

PDR: proliferative diabetic retinopathy; IVC: intravitreal conbercept injection + pars plana vitrectomy; No-IVC: pars plana vitrectomy only; Control-MH: control group was patients with macular holes; VEGF-A: vascular endothelial growth factor A. * *p* value < 0.05.

**Table 3:** Comparison of bleeding scores between the two groups ($\bar{x}$ ± s).

Group	PDR		VH		VH + FVM	
	*n*	HS	*n*	HS	*n*	HS
IVC	124	1.27 ± 0.73	26	0.88 ± 0.78	98	1.36 ± 0.69
No-IVC	28	1.46 ± 0.84	13	1.07 ± 0.86	15	1.80 ± 0.68
*p* value	—	0.665	—	0.498	—	0.026 *

PDR: Proliferative diabetic retinopathy; VH: vitreous hemorrhage; FVM: fibrovascular membrane; HS: hemorrhage score. * *p* value < 0.05.

**Table 4:** Bleeding analysis for PDR complexity subgroup (*n*/%).

	SC < 3			SC ≥ 3		
	*n*	Hemorrhage (*n*/%)	No Hemorrhage (*n*/%)	*n*	Hemorrhage (*n*/%)	No Hemorrhage (*n*/%)
IVC	82	31/37.8	51/62.2	42	21/50.0	21/50.0
No-IVC	18	8/44.4	10/55.6	10	9/90.0	1/10.0
*p* value	—	0.601		—	0.032 *	

SC: Complexity score; n: number. * *p* value < 0.05.

**Table 5:** Comparison of postoperative rebleeding rate between the two groups.

Group	1 Month		3 Month	
	*n*	*n* (%)	*n*	*n* (%)
IVC	116	7 (6.03)	109	6 (5.50)
No-IVC	26	6 (23.08)	25	1 (4.00)
*p* value	—	0.012 *	—	0.361

IVC: Intravitreal conbercept injection + pars plana vitrectomy; No-IVC: pars plana vitrectomy only. * *p* value < 0.05.

**Table 6:** The number of patients with ocular hypertension.

Group	Preoperative		1 Week		1 Month		3 Month	
	*n*	High IOP (*n*)	*n*	High IOP (*n*)	*n*	High IOP (*n*)	*n*	High IOP (*n*)
IVC	121	8	120	14	116	20	109	11
No-IVC	28	1	26	1	26	2	22	1
*p* value	—	0.866	—	0.404	—	0.359	—	0.676

IVC: Intravitreal conbercept injection + pars plana vitrectomy; No-IVC: pars plana vitrectomy only; High IOP: high intraocular pressure; *n*: number.

**Table 7:** NVG at 2 years after surgery between two groups.

Group	PDR (*n*)	NVG (*n*)	*p*
IVC	111	13	0.736
No-IVC	26	2	

IVC: Intravitreal conbercept injection + pars plana vitrectomy; No-IVC: pars plana vitrectomy only.

## Data Availability

Data are contained within the article.
